# On-Road Evaluation of Unobtrusive In-Car Respiration Monitoring

**DOI:** 10.3390/s24144500

**Published:** 2024-07-11

**Authors:** Adrian Radomski, Daniel Teichmann

**Affiliations:** SDU Health Informatics and Technology, The Maersk Mc-Kinney Moller Institute, University of Southern Denmark, 5230 Odense, Denmark; date@mmmi.sdu.dk

**Keywords:** unobtrusive monitoring, breathing rate, vital signs, continuous health monitoring, automotive sensors

## Abstract

This paper introduces and evaluates an innovative sensor for unobtrusive in-car respiration monitoring, mounted on the backrest of the driver’s seat. The sensor seamlessly integrates into the vehicle, measuring breathing rates continuously without requiring active participation from the driver. The paper proves the feasibility of unobtrusive in-car measurements over long periods of time. Operation of the sensor was investigated over 12 participants sitting in the driver seat. A total of 107 min of driving in diverse conditions with overall coverage rate of 84.45% underscores the sensor potential to reliably capture physiological changes in breathing rate for fatigue and stress detection.

## 1. Introduction

In recent years, the integration of health monitoring systems into vehicles has gained significant attention as a means to address the challenges posed by modern healthcare systems while enhancing driver safety and well-being. Among the physiological parameters monitored, respiration rate is a crucial indicator of overall health and driver condition [1]. Accurate monitoring of respiration can provide insights into a driver’s physical and emotional state [2], reflecting levels of stress [3], fatigue [4], and alertness [5]. Traditional methods for monitoring respiration typically involve direct contact with the skin, using devices such as chest straps or nasal cannulas [6]. These methods, while effective, are often intrusive and impractical for continuous use in a driving environment.

Therefore, there is a need for a noninvasive, unobtrusive solution that can seamlessly integrate into the vehicle and provide continuous monitoring without causing discomfort or distraction to the driver.

Non-contact respiration monitoring systems, which do not require physical contact, can be divided into acoustic [7], radar [8], optical [9,10], and thermal methods [11]. Each has unique benefits and challenges. Acoustic systems utilize Doppler shifts in exhaled breaths but are sensitive to environmental noise and patient movement. Radar methods, like FMCW [12] and UWB [13] radars, detect chest movements but face accuracy issues with spontaneous breathing and radio frequency exposure concerns. Optical methods, using cameras to track chest wall movements, are costly and affected by lighting and motion artifacts [14]. Thermal methods detect temperature changes from exhaled air but require close proximity to the patient and are slow to process [15].

The magnetic coupling-based method presented in this paper offers distinct advantages over other non-contact methods. This approach enables seamless integration into the vehicle environment, providing continuous and unobtrusive monitoring without requiring active participation from the driver. Unlike acoustic systems, it is less susceptible to environmental noise. Compared to radar methods, it does not pose concerns related to radio frequency exposure, and can maintain accuracy regardless of spontaneous breathing patterns. Additionally, it avoids the high costs and sensitivity to lighting conditions associated with optical methods, and it does not necessitate slow processing like thermal methods. All of this makes the magnetic coupling-based method a robust choice for in-car respiration monitoring.

In this study, we introduce and evaluate a novel magnetic induction (MI) sensor designed for unobtrusive in-car respiration monitoring. Unlike existing non-contact methods, our sensor utilizes magnetic fields to detect breathing rate changes and is mounted on the backrest of the driver’s seat, enabling operation through clothing.

Previous research has explored MI sensors [16,17,18], but this paper uniquely demonstrates their feasibility for prolonged driving monitoring and for measuring respiration rates over larger populations. Additionally, we employ a different size and winding number for the coil, a crucial parameter of the sensor, compared to other studies.

The primary objective is to assess the sensor’s accuracy and reliability in real-world driving conditions with 12 participants in an idle state and to demonstrate its feasibility during prolonged drives with a single subject. For the prolonged drive, we conducted two rides totaling 107 min to determine if the sensor can effectively overcome motion artifacts, a common challenge for all non-contact sensors. This phase included diverse driving conditions such as city traffic, rural roads, and highways to test the sensor’s sensitivity to noise across various environments. This investigation aims to provide a practical solution for continuous respiration monitoring in vehicles, contributing to the advancement of driver assistance systems and enhancing road safety.

The following Methods section dives into the theory of operation and the experimental design, while the Results section presents the measurements obtained during the study.

## 2. Methods

### 2.1. Theory of Operation

**Figure 1:**
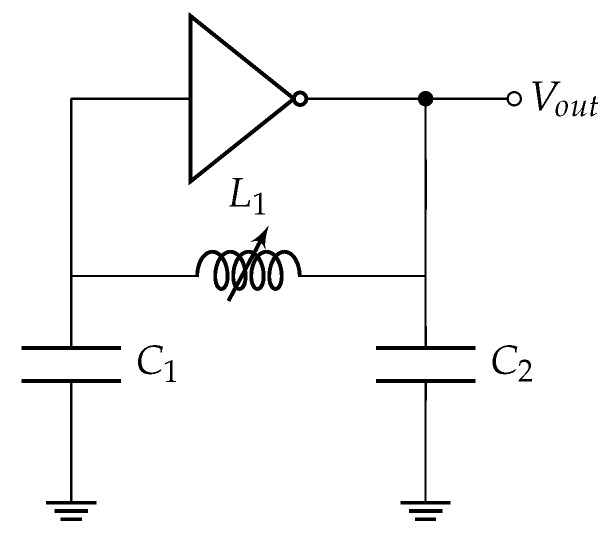
Schematic of the Colpitts CMOS Oscillator circuit of the MI sensor.

The core component of the MI sensor is a circuit based on a CMOS Colpitts Oscillator. The circuit is depicted in Figure 1.

**Figure 2:**
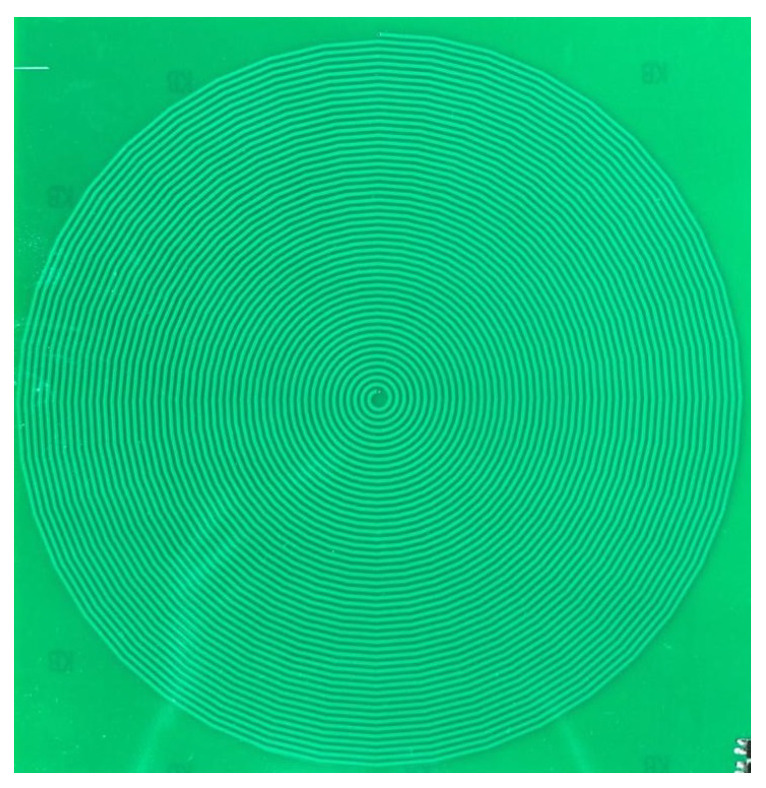
Photograph of the coil of the MI sensor.

This oscillator includes a coil, two capacitors, and an inverter, all working together to meet the Barkhausen criteria for sustained oscillations. The circuit’s capacitances $C_{1}$ and $C_{2}$ are each 10 pF, while the coil, possesses an inductance of $L_{1}=177.84\;\mu H$. The resistance of the coil is 10.76Ω. The diameter of the coil is 20 cm and the coil has 50 windings. The width of the wire is 1 mm and the spacing between the windings is also 1 mm. The oscillator operates at a frequency of 2.4 MHz, with the coil being the varying—and hence, frequency modulating—parameter of the sensor. A photograph of the coil is presented in Figure 2.

When alternating current flows through the coil, it generates a primary magnetic field. This magnetic field interacts with the driver’s body, inducing eddy currents within the body tissues. These eddy currents, in turn, generate their own magnetic field, known as the secondary magnetic field. The interaction between the primary and secondary magnetic field affects the reflective impedance of the coil [18].

The secondary magnetic field varies with changes in the bioimpedance of the driver’s body, which is influenced by the respiration rate. As the driver breathes, the bioimpedance fluctuates due to the expansion and contraction of the chest, altering the characteristics of the secondary magnetic field. These changes in the secondary magnetic field modify the reflective impedance of the coil.

The CMOS Colpitts Oscillator is sensitive to these impedance changes. As the reflective impedance of the coil changes, the oscillator’s parameters are affected, leading to variations in the frequency of the current flowing through the coil. These frequency shifts are directly proportional to the changes in the reflective impedance of the coil, which in turn are influenced by the driver’s breathing rate. The oscillator generates a square wave output, which is then fed into a microcontroller for quantization. The microcontroller counts the number of pulses within a specified time window, converting the frequency shifts into a digital signal that can be monitored.

### 2.2. Setup

**Figure 3:**
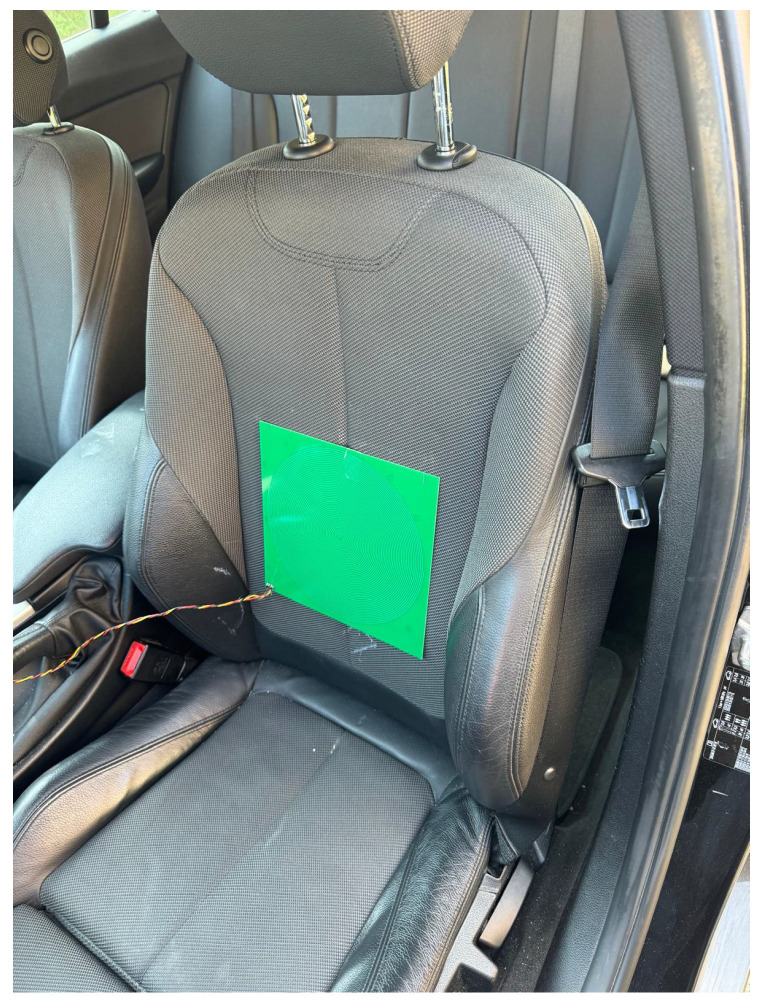
Photograph of the MI sensor postioned on the driver seat’s backrest in a BMW 320 car.

The MI sensor for unobtrusive in-car respiration monitoring was installed onto the backrest of the driver’s seat. Figure 3 illustrates the spatial arrangement of the sensor within the car seat. For reference, a strain gauge sensor wrapped around the chest of the subjects was used. The strain gauge sensor, considered a gold standard in respiration monitoring (Biopac Bionomadix BN-RSP2-T) was connected to the MP160 system from Biopac Systems Inc. (Goleta, CA, USA). The acquisition sampling rate for the reference system was set at 2 kS/s with 16 bit.

The investigated sensor, unlike the Biopac system, generated a digital signal. The output of this sensor was the frequency of the oscillator. The frequency was calculated using an Arduino system, employing a standard peak detection method. Peaks were detected using a 33 ms window, within which all peaks were counted. The output from the Arduino was connected via a UART-to-USB adapter to a computer, where data were logged using CoolTerm software (version 2.1.1).

**Figure 4:**
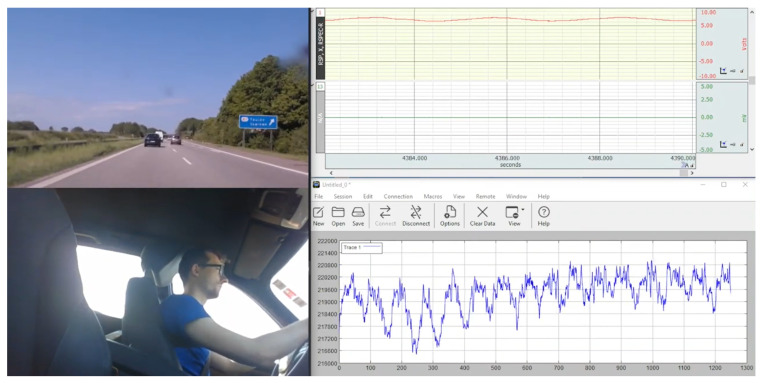
Acquired data during on-road measurement: street view, driver view, and sensor signals ((**top**) gold standard (**bottom**) custom sensor signal).

To establish links between sensor data and driving behaviors, two cameras were installed within the cockpit. One camera was mounted to capture the street view through the windshield, while the other one focused on the subject. An exemplary snapshot of the camera views and the recorded data can be seen in Figure 4.

### 2.3. Measurement Scenarios and Protocol

The study comprises two distinct parts, each designed to comprehensively evaluate the performance of the sensor in varying driving conditions.

#### 2.3.1. Validation of Sensor Performance

In the first part of the study, a population study was conducted involving 12 participants, comprising 8 men and 4 women. This initial phase focused on assessing the sensor’s general functionality under idle conditions. Participants were seated in the driver’s seat with the sensor installed, and data were collected to evaluate the sensor’s baseline performance.

Participants provided informed consent, demonstrating their understanding of the study’s objectives and protocols. The participant cohort consisted of healthy individuals spanning ages 21 to 29 years. Throughout the experiments, participants adhered to a single clothing layer to maintain consistency in the sensor’s measurements and minimize potential confounding variables.

#### 2.3.2. On-Road Evaluation

The on-road evaluation consisted of two driving sessions: a 52 min drive and a 57 min drive. During these sessions, the participant was tasked with navigating diverse road conditions, including city driving, rural routes, and highway stretches. This setup allowed for the assessment of the sensor’s performance in real-world driving scenarios, considering factors such as road noise, vibrations, and varying driving behaviors. The first drive focused on highway conditions, while the second drive concentrated on rural routes.

### 2.4. Data Processing and Statistical Methods

Recorded data were processed using Matlab 2023a to accurately extract the breathing rate signal. A two-stage processing method was employed, ensuring robustness and reliability in the analysis.

The signal underwent band-pass filtering within the frequency range of 0.15 Hz to 0.50 Hz to isolate the breathing rate signal from noise and artifacts. Subsequently, peak identification was conducted using Matlab’s findpeaks function. Peaks in the signal were identified, indicating individual breaths. Following peak extraction, the average peak-to-peak time was computed within a sliding window of 120 s length, with a 10 s overlap. This provided a measure of the breathing rate over time.

The rationale behind choosing a 120 s window was to balance between capturing short-term variations and minimizing noise. Breathing rates can vary over short periods due to various factors, such as minor physical movements or changes in breathing patterns. Averaging over a 120 s window helps to smooth out these short-term fluctuations, providing a more stable and reliable measure of the breathing rate. Additionally, this duration is long enough to include multiple breathing cycles, ensuring that the calculated average is representative of the overall breathing pattern while still being responsive to longer-term trends.

Evaluation of sensor accuracy was conducted using Bland–Altman plots, a widely used method for assessing agreement and potential biases between measurements. Each plot visually illustrates the mean difference between the arithmetic mean of the sensor reading and the reference reading on the horizontal axis, while the vertical axis represents the measurement error, calculated as the difference between the reference system and the MI system readings: [ReferenceSystem]−[MISystem].

The plots display the mean difference along with the mean ± 1.96 times the standard deviation, corresponding to a 95% confidence interval. This detailed visualization helps to identify typical biases and potential disturbances such as signal loss due to maneuvers or specific noise from road conditions that may cause deviations, particularly in long interval regions.

To establish a clear benchmark for sensor accuracy, a predetermined acceptable error margin of 5% was set. This allowed for the identification of any discrepancies between the sensor readings and the reference readings, ensuring the reliability and validity of the sensor’s measurements.

## 3. Results

### 3.1. Validation of Sensor Performance

**Figure 5:**
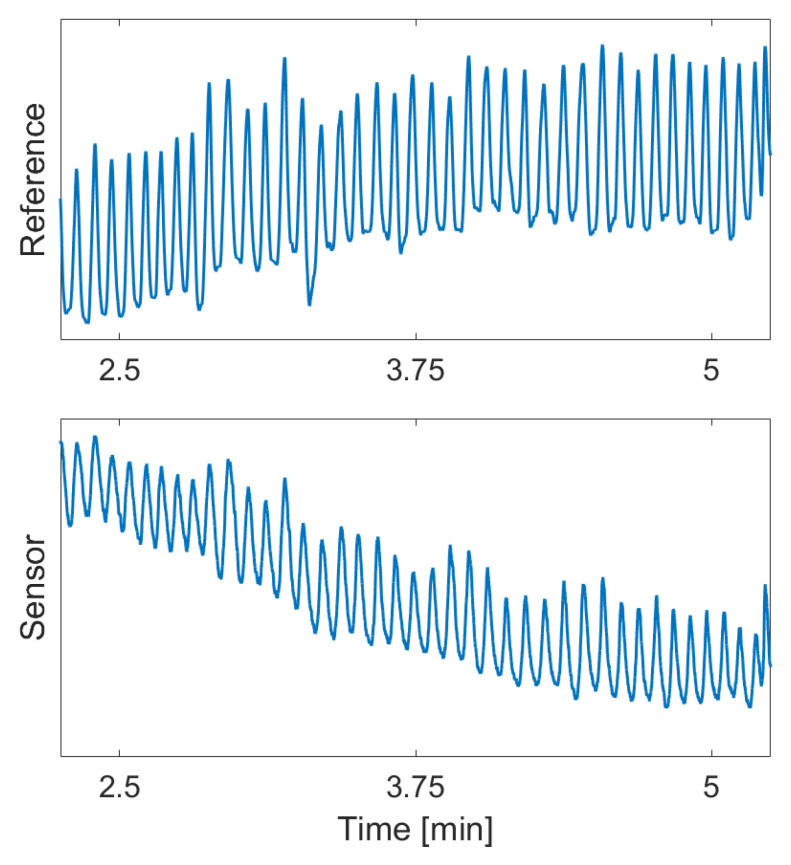
Exemplary excerpt of the signal and the reference breathing rate in idle conditions.

An exemplary sensor signal captured during idle sitting conditions is illustrated in Figure 5, alongside the corresponding reference signal.

The graphs illustrate the data captured over a period of 3 min.

**Figure 6:**
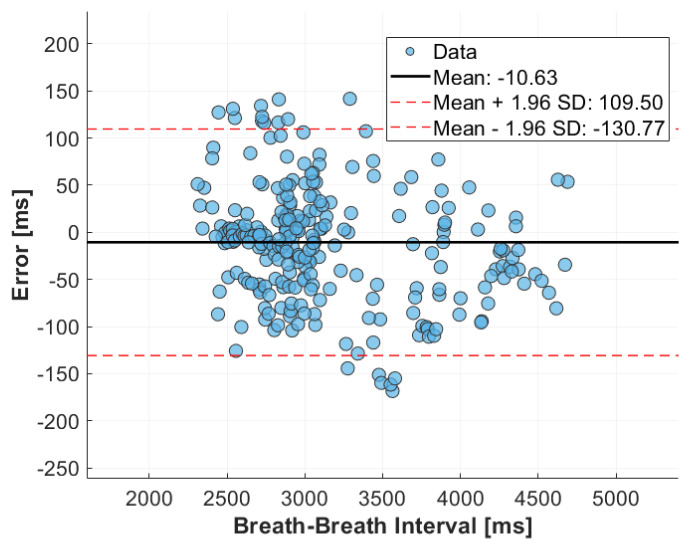
Bland-Altman plot over all twelve subjects comparing breath-to-breath intervals derived from the MI sensor and reference breathing rate monitor during driving.

Figure 6 illustrates the Bland–Altman plot comparing the breath-to-breath times derived from the MI sensor with that extracted from the reference strain gauge sensor. The plot comprises 292 data points, collected from all 12 subjects. Analysis of the data reveals a mean difference in breath-to-breath time identification of −10.63 ms, indicating minimal systematic bias between the sensor readings and the reference measurements. Furthermore, the limits of agreement, with an upper limit of 109.50 ms and a lower limit of −130.77 ms, demonstrate low deviation from the reference over the entire experiment.

### 3.2. On-Road Evaluation

Table 1 shows the coverage rates of the continuous breathing rate data during both driving sessions, categorized by the percentage of data falling below various error thresholds. Overall, the sensor maintained a high coverage rate, with 84.45% of the data falling below a 5% error threshold. Notably, highway drive exhibited significantly higher coverage rates across all error thresholds compared to rural drive. Specifically, 96.81% of the data from highway drive had an error rate below 5%, while only 72.08% of the data from rural drive met this criterion. This trend was consistent across lower error thresholds as well, with highway drive showing superior performance.

#### 3.2.1. Highway Drive

**Figure 7:**
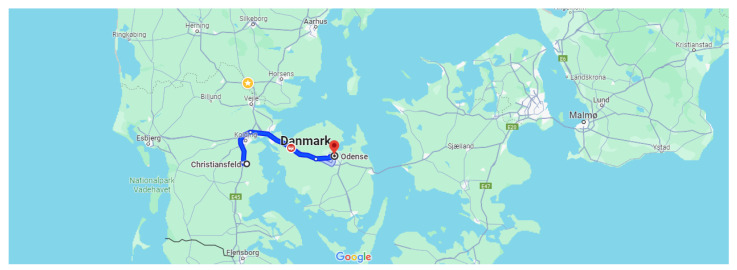
Driving route—highway drive.

Highway drive encompassed a route from Nørre Abby towards Middelfart, initially traversing rural roads before entering the city. The driver then proceeded onto the highway and continued towards Christiansfeld. The driving route is illustrated in Figure 7. During this drive, the speed was typically maintained at 120 km/h, with occasional peaks reaching up to 160 km/h.

**Figure 8:**
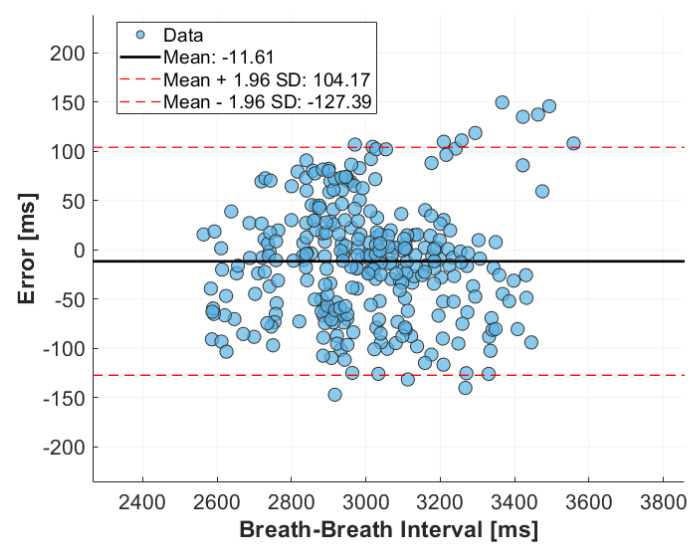
Bland-Altman plot comparing breath-to-breath intervals derived from the MI sensor and reference breathing rate monitor during highway drive.

The accuracy of the MI sensor during this drive was evaluated using Bland–Altman analysis, as shown in Figure 8. The Bland–Altman plot demonstrates that the sensor data closely followed the reference data, with limits of agreement ranging from −127.39 ms to 104.17 ms and a mean error of −11.61 ms. These limits of agreement constitute 3.47% of the mean signal, indicating a high level of agreement between the sensor and the reference standard.

**Figure 9:**
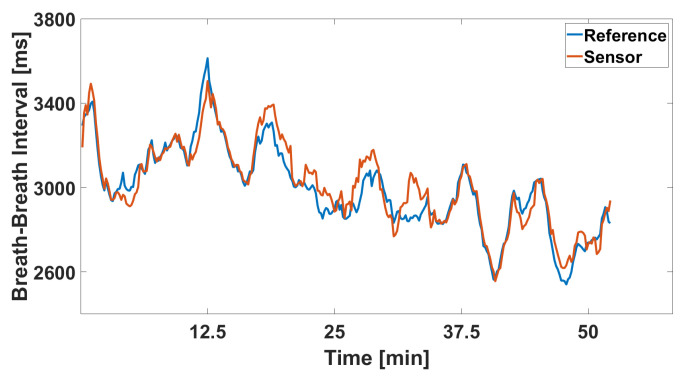
Extracted breath-to-breath intervals over the entire highway drive.

Additionally, the signal versus reference signal plot, depicted in Figure 9, provides a time-based comparison of the recorded breathing rate against the reference standard. The plot reveals that the monitored signal aligns closely with the gold standard method throughout the drive. The fluctuations in breathing rate are clearly visible, and the sensor accurately captures these changes. Notably, the data indicate a gradual increase in breathing rate, which may suggest the onset of driver fatigue.

#### 3.2.2. Rural Drive

**Figure 10:**
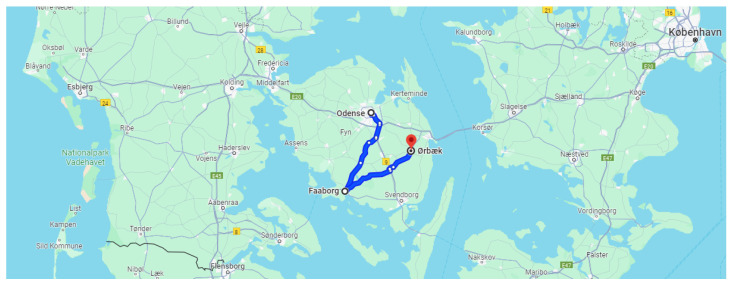
Driving route—rural drive.

Rural drive consisted of a journey from Odense to Faaborg, initially following a highway route before transitioning to rural roads, which continued until the end of the drive in Ørbaek. The driving route is visually represented in Figure 10. During this drive, the speed was typically around 90 km/h, except when driving in populated areas where the speed was reduced to approximately 50 km/h.

**Figure 11:**
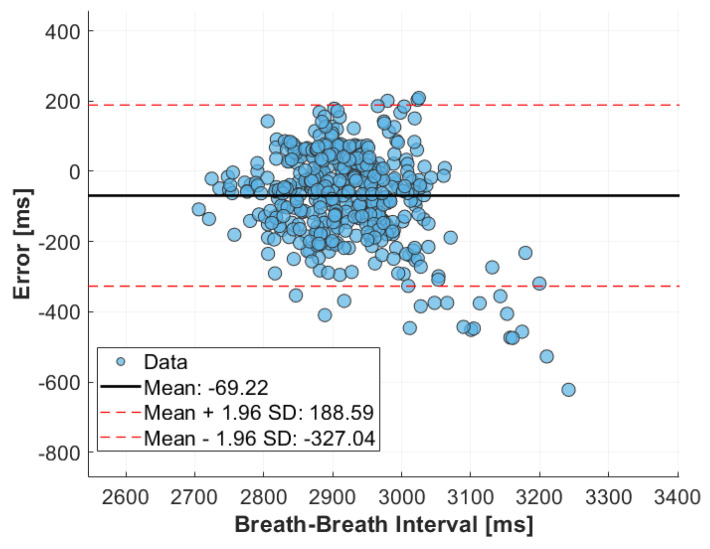
Bland-Altman plot comparing breath-to-breath intervals derived from the MI sensor and reference breathing rate monitor during rural drive.

The accuracy of the MI sensor during rural drive was assessed using Bland–Altman analysis, as shown in Figure 11. The Bland–Altman plot reveals a mean error of −69.22 ms, with limits of agreement ranging from −327.04 ms to 188.56 ms. These limits of agreement represent 8.59% of the mean breath length, indicating a significant increase in error compared to highway drive. The plot also shows that measurements of breath-to-breath values higher than 3000 ms have a considerably larger error, while values smaller than 3000 ms exhibit much smaller errors.

**Figure 12:**
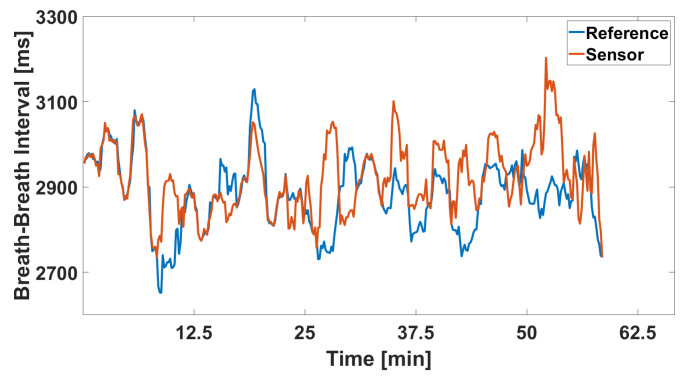
Extracted breath-to-breath intervals over the entire rural drive.

Additionally, the signal versus reference signal plot, depicted in Figure 12, provides a time-based comparison of the recorded breathing intervals against the reference standard. While the graphs do not exhibit significant errors, it is challenging to discern a clear trend from the investigated signal. The alignment with the reference signal is less consistent compared to highway drive, reflecting the increased variability and potential inaccuracies in the sensor data during rural road conditions.

The results from rural drive indicate that the in-car respiration monitoring sensor’s performance is less reliable on rural roads compared to highway conditions. The higher mean error and wider limits of agreement highlight the increased difficulty in maintaining accurate readings under these variable driving conditions, despite the lack of significant errors in the time-based comparison.

### 3.3. Influence of Driving Situation on Signal Quality

After presenting the overall data from both rides, it is essential to investigate how the signal behaves under various road conditions such as turns, traffic lights, changes in driving environment, and the differences between rural roads and highways. This section aims to analyze the signal quality in different driving scenarios to understand the sensor’s performance comprehensively.

#### 3.3.1. Engine Idle vs. Highway Drive

**Figure 13:**
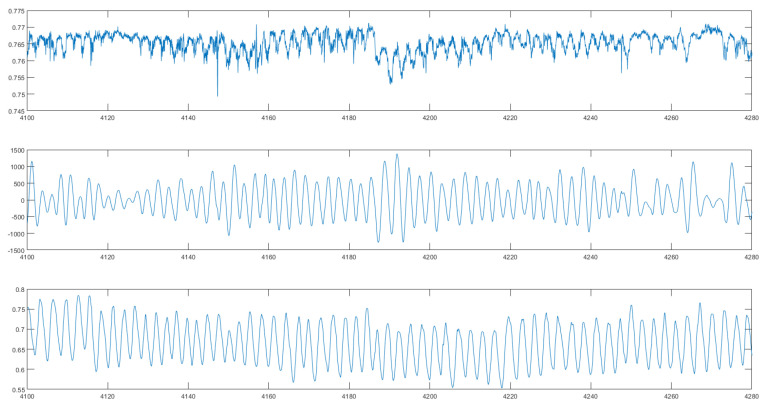
The signal of the unobtrusive respiration signal while driving. (**Upper diagram**): raw signal; (**middle diagram**): filtered signal; (**lower diagram**) reference signal.

First, we compare the signal changes when measured in an idle state with the engine on versus during a highway drive. Figure 13 displays two charts. The top chart shows the unfiltered signal from the highway over a span of five minutes, while the graph below demonstrates how a filter with a bandwidth of 0.15Hz to 0.5Hz effectively eliminates highway noise. It is evident that the high level of noise from the highway is significantly reduced by the tight boundaries of the filter, resulting in a cleaner signal.

**Figure 14:**
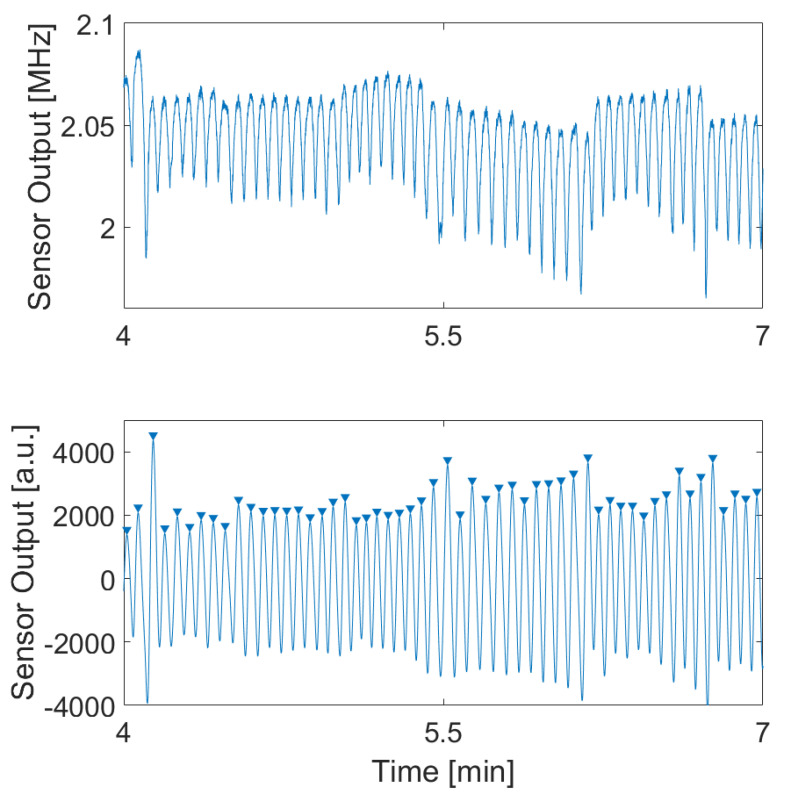
The signal of the unobtrusive respiration signal with engine in idle mode. (**Upper diagram**): raw signal; (**lower diagram**): filtered signal.

Figure 14 illustrates the signal behavior in an idle state. The unfiltered signal in this state is of much higher quality compared to the highway drive, with clearly visible peaks that require minimal filtering. The filtered signal in the idle state is significantly higher in quality than the filtered signal from the highway, indicating that the sensor operates more accurately when the vehicle is stationary.

#### 3.3.2. Rural Drive vs. Highway Drive

**Figure 15:**
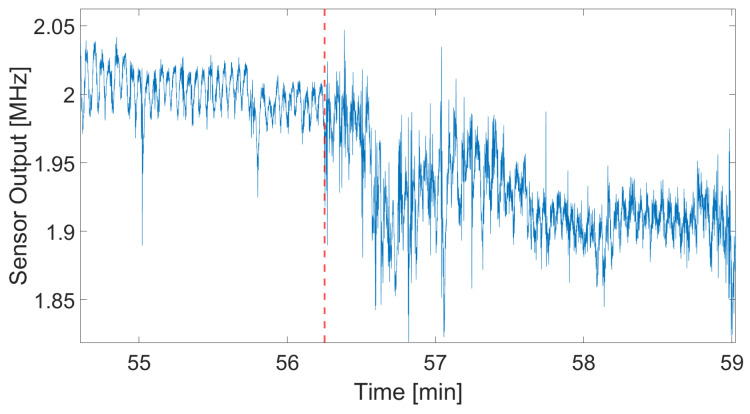
Effect of road conditions on the sensor signal. The red line indicates the transition from rural road to highway.

Next, we examine the signal quality during a transition from rural roads to the highway. Figure 15 presents a graph depicting this transition. The red dashed line represents the transition from rural ride to highway. The rural road section is characterized by much lower noise levels compared to the highway, which is attributable to the lower speeds typical of rural driving. In contrast, highway driving involves higher speeds and, consequently, more noise.

During the transition period, it becomes challenging to measure the breathing rate accurately due to the varying noise levels. However, once the driver enters the highway and maintains a steady speed, the signal stabilizes, and the noise levels decrease. This observation highlights the sensor’s sensitivity to motion artifacts, which significantly impact signal quality during changes in driving conditions.

#### 3.3.3. Deceleration, Stopping, and Starting at Traffic Lights

**Figure 16:**
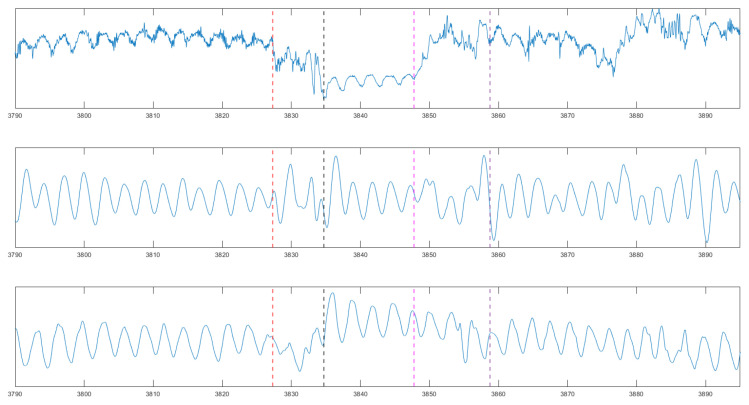
The signal of the MI sensor over the course of one stop and start. red: start decelaration; black: total stop; magenta: start of acceleration; violet: end of acceleration. (**Upper diagram**): raw signal; (**middle diagram**): filtered signal; (**lower diagram**): reference signal.

In this subsection, we analyze the behavior of the sensor during phases of deceleration, stopping, and acceleration at traffic lights. The graph in Figure 16 illustrates the signal during these different phases in a city driving scenario. The red dashed line represents the beginning of the deceleration, the black dashed line represents stopping, the magenta dashed line represents starting, and the violet line represents the end of the acceleration.

The graph starts by showing the signal while driving in the city. This phase demonstrates typical urban driving conditions, with the sensor capturing the breathing rate amidst the moderate noise associated with city traffic. As the driver approaches a traffic light and begins to decelerate, the signal becomes more challenging to interpret due to motion artifacts introduced by the slowing down of the vehicle. These artifacts make it difficult to accurately measure the breathing rate during the deceleration phase.

When the vehicle comes to a stop at the traffic light, the motion artifacts diminish, and the sensor can more easily read the breathing rate. The signal during this idle phase is significantly clearer, allowing for more precise respiration monitoring. This phase is contrasted with the signal during city driving, highlighting the improved signal quality when the vehicle is stationary.

As the traffic light turns green, the driver accelerates, reintroducing motion artifacts that once again complicate the measurement of the breathing rate. However, once the vehicle reaches a steady speed and the motion artifacts settle, the quality of the signal returns to that of the initial driving phase. This demonstrates the sensor’s capability to recover and provide accurate readings once the driving conditions stabilize.

#### 3.3.4. Turning

**Figure 17:**
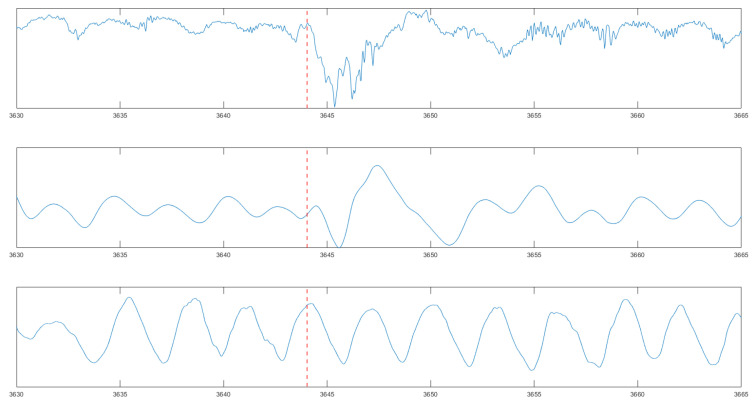
The signal of the MI sensor over the course of one turn. The start of turn is indicated by the red line. (**Upper diagram**): raw signal, (**middle diagram**): filtered signal, (**lower diagram**): reference signal.

In this final subsection, we examine the sensor’s behavior during turning maneuvers. Figure 17 displays the signal captured during a turn, highlighting the impact of motion artifacts on the sensor’s ability to accurately monitor the breathing rate. The red dashed line represents the beginning of turning.

The graph shows that during the turning phase, the signal is significantly affected by motion artifacts, making it challenging for the sensor to provide accurate breathing rate measurements. The physical movement associated with turning introduces noise and instability in the signal, complicating the monitoring process.

However, once the turn is completed and the driver resumes a more stable position with minimal movement, the quality of the signal improves. This post-turn phase allows the sensor to again accurately measure the breathing rate, as the motion artifacts diminish and the signal stabilizes.

## 4. Discussion

The successful implementation and evaluation of the MI sensor presented in this study underscore its potential for real-world application. Our findings demonstrate that the sensor, integrated into the driver’s seat backrest, can effectively track the driver’s breathing rate without requiring active involvement. The coverage rate of 84.45% achieved over 107 min of driving across various conditions, including city drives, rural routes, and highways, highlights the sensor’s robustness and adaptability to diverse driving environments.

However, our study also revealed challenges associated with motion artifacts, which significantly influence the sensor’s performance. The sensor’s sensitivity to driver movements, particularly during turning, acceleration, and deceleration phases, poses difficulties in accurately monitoring breathing rates. Despite the effectiveness of tight filters in minimizing external noise, such as speed-related disturbances, additional strategies are needed to address motion artifacts effectively.

Future research directions may involve enhancing the sensor’s magnetic field strength and exploring novel approaches for determining the oscillatory frequency to improve signal quality [19]. Moreover, investigating alternative filtering and noise detection methods could help mitigate the impact of motion artifacts on sensor performance during driving maneuvers.

Notably, our feasibility study identified the potential utility of the sensor for fatigue and stress detection, as evidenced by the observed trend in one of the driving sessions. However, it is essential to acknowledge the limitations of our study in this regard, particularly the small sample size. Future studies should aim to replicate our findings with a larger and more diverse group of participants to ensure the generalizability of the results.

Our comparison between driving environments revealed that while highways present fewer challenges for the sensor due to smoother driving conditions and easier noise filtration, rural roads pose greater difficulties. The increased frequency of driver movements and turns on rural roads complicates breathing rate monitoring, despite the relatively lower speed-related noise. This disparity is evident in the variation in coverage rates between highway drive (96.81%, mainly highways) and rural drive (72.08%, mainly rural roads). Notably, errors in rural road measurements can be readily identified, as measurements above a certain threshold correlate with higher error rates.

Furthermore, the comparison between idle measurements and driving environments highlights the impact of driving-related noise on signal deterioration. The significant degradation in signal quality during driving underscores the need for continued research into noise mitigation strategies to optimize sensor performance in dynamic driving conditions.

## 5. Conclusions

This paper evaluates an innovative in-car respiration monitoring sensor designed to seamlessly integrate into the driver’s seat, offering unobtrusive monitoring of breathing rates during driving. Through rigorous validation and on-road evaluation, the sensor demonstrated commendable accuracy and reliability in tracking breathing rate signals across various driving conditions, including city drives, rural routes, and highways. The results of the study highlight the sensor’s robust performance, with an impressive coverage rate of 84.45% achieved over 107 min of driving, underscoring its potential for real-world application in fatigue and stress detection.

## Figures and Tables

**Table 1:** Coverage rates of the MI sensor during driving.

	Percent of Data Below Error Threshold				
	**< 5%**	**< 4%**	**< 3%**	**< 2%**	**< 1%**
Overall	84.45%	77.43%	68.33%	48.18%	29.74%
Highway Drive	96.81%	93.29%	82.43%	59.74%	41.85%
Rural Drive	72.08%	61.56%	54.23%	36.61%	17.62%

## Data Availability

The data supporting the reported results can be found in the following YouTube videos: Rural Drive Dataset (https://www.youtube.com/watch?v=v7Txr3LTffA, accessed on 19 June 2024) and Highway Drive Dataset (https://www.youtube.com/watch?v=ap2FMvcp8SM, accessed on 19 June 2024).
